# Discovery of Post‐Translationally Modified Epitopes in Autoimmunity: Someone Has to Fish so Everyone Can Eat

**DOI:** 10.1111/imr.70179

**Published:** 2026-09-27

**Authors:** Sanya Arshad, Benjamin M. Cameron, Alok V. Joglekar

**Affiliations:** ^1^ Center for Systems Immunology University of Pittsburgh School of Medicine Pittsburgh Pennsylvania USA; ^2^ Departments of Immunology and Computational Systems Biology University of Pittsburgh School of Medicine Pittsburgh Pennsylvania USA; ^3^ Graduate Program in Microbiology and Immunology University of Pittsburgh School of Medicine Pittsburgh Pennsylvania USA; ^4^ Cancer Immunology and Immunotherapy Program UPMC Hillman Cancer Center USA

## Abstract

Post‐translational modifications (PTMs) chemically diversify the ~20,000 genomically encoded proteins into millions of functional variants, and this diversity has profound consequences for T cell‐mediated immune responses. PTM‐reactive T cells have been identified across numerous autoimmune diseases and, in several cases, implicated directly as pathogenic drivers. Mechanistically, PTMs promote autoimmunity through two intertwined routes: by altering peptide–MHC and TCR binding to favor recognition of self‐epitopes, and by generating neoepitopes in peripheral and/or inflamed tissues that are absent from the thymus, allowing PTM‐reactive T cells to escape central tolerance. This cascade is amplified by inflammatory cytokines, upregulated antigen‐processing machinery, disease‐associated HLA alleles, and, in some cases, exogenous triggers such as diet. We use three well‐characterized examples—citrullination in multiple autoimmune disorders, hybrid insulin peptide (HIP) formation in type 1 diabetes, and gluten deamidation in celiac disease—to illustrate how distinct PTM biology, tissue specificity, and genetic encodability shape the antigenic landscape and dictate feasible discovery strategies. We then review four complementary approaches for identifying PTM‐specific T cell responses: mass spectrometry‐based proteomics and immunopeptidomics, computational MHC‐binding prediction, antigen‐directed methods, and high‐throughput T cell receptor (TCR)‐directed library screening methods. For each, we discuss underlying principles, strengths, and limitations, with particular attention to the challenge posed by non‐canonical, PTM‐bearing residues. By comparing citrullination, HIPs, and gluten deamidation across these methodologies, we highlight how biochemical alterations, tissue accessibility, and mouse‐to‐human conservation determine which discovery approach is most tractable. We conclude that while synthetic peptide‐based methods remain the standard for validating known epitopes, emerging high‐throughput, cell‐based platforms are rapidly expanding the accessibility of the PTM antigenic landscape for both known and undiscovered autoimmune epitopes.

## 1 Antigenic Landscape of T Cell Responses

T cells are critical components of the immune system, protecting against infections and cancers, but causing autoimmunity if dysregulated. T cells use their surface T cell receptor (TCR) to mount antigen‐specific responses [1]. TCRs can recognize peptide and non‐peptide epitopes presented by classical or non‐classical major histocompatibility complex (MHC) molecules (also called human leukocyte antigen (HLA) molecules in humans) [2]. For the purposes of this review, we will focus on T cells carrying α/β TCRs that recognize linear peptide epitopes presented on classical MHC/HLA. CD8+ T cells recognize epitopes in class I MHC which are found on virtually all nucleated cells, whereas CD4+ T cells recognize epitopes in class II MHC found on professional antigen‐presenting cells (APCs). Class I MHC molecules sample the intracellular proteome by presenting translated peptides, whereas class II MHC molecules sample the extracellular proteome by presenting peptides that are endocytosed [3]. However, there is a significant amount of cross‐talk between these presentation pathways under homeostatic and inflammatory conditions [4]. It is estimated that 10^4^–10^5^ class I molecules are presented on the surface of a single cell, whereas a professional APC can present 10^5^–10^6^ class II molecules [5]. This amounts to 10^5^–10^6^ unique peptide epitopes being presented on a cell. T cell responses deal with this diversity by generating a large diversity of unique TCRs through V(D)J recombination. TCR repertoire estimates vary widely, but are usually thought to be in the range of 10^6^–10^9^ unique TCRs in a given individual's immune system [6, 7, 8]. This combinatorial diversity of TCRs and pMHC complexes is immensely beneficial from a standpoint of ensuring protection against a broad range of threats, but does pose significant challenges in studying the immune system.

Single‐cell and bulk TCR sequencing methods have made significant advances in being able to profile TCR repertoires in mice and humans. Concomitantly, proteomics and immunopeptidomics methods have advanced our understanding of the landscape of antigens that are presented in MHC. Finally, several methods have been developed to connect these two facets in order to identify antigen‐specific T cell responses. These methods are faced with a formidable challenge—the antigenic landscape is enormous, highly diverse, and dynamic. Adding to this challenge is the fact that proteins undergo post‐translational modifications (PTMs). PTMs are chemical modifications of amino acid side chains that alter proteins after synthesis. A wide variety of modifications including phosphorylation, glycosylation, methylation, acetylation, and ubiquitination are added to proteins throughout their life cycle [9, 10, 11, 12]. PTMs can change the structural, biophysical, and biochemical properties of proteins, often providing chemical moieties crucial for their function and regulation [13]. PTMs can be incorporated into proteins through chemical, non‐enzymatic processes, for instance oxidation [14, 15] and carbamylation [16, 17], by reactive metabolites and cellular stress. Alternatively, specific enzymes can confer PTMs such as methylation [18, 19, 20], acetylation [21, 22, 23], and citrullination [24, 25] under homeostatic or inflamed conditions [26, 27, 28, 29]. Overall, PTMs are critical for modulating protein structure, function, homeostasis, stability, and regulation. Importantly, PTMs expand the cellular proteome from ~20,000 genomically encoded proteins to millions of functional protein variants.

A significant consequence of PTMs is their altered immunogenicity. The fact that PTM‐specific immune responses are present in autoimmunity, cancers, and beyond is well established [30, 31]. While modified proteins are targets of both humoral and cellular responses, in this review we will focus exclusively on T cell responses to PTMs. PTM‐reactive T cells have been detected across many autoimmune diseases, and in some cases have been implicated as drivers of pathogenicity [32, 33, 34]. While the literature showing antibodies or T cells recognizing PTMs is vast, a concise and non‐exhaustive list of PTMs relevant in autoimmunity is summarized in Table 1. Mechanistically, PTMs can drive autoimmunity through two routes: (1) altering the binding of epitopes to MHC and/or TCR, thereby increasing the likelihood of stimulating T cells that would have recognized the unmodified epitopes [68, 69, 70, 71, 72] (Figure 1); and (2) increasing the likelihood of T cells reactive to PTMs escaping central tolerance, as PTMs are often induced in peripheral tissues under conditions of stress and/or inflammation and are absent from the thymus [73, 74, 75]. Taken together, this leads to a cascade where a PTM is generated under inflammation in the periphery, leading to a more immunogenic epitope presented on the inflamed tissue, which is recognized by the T cell responses sensing the modified epitope as a neo‐epitope and attacking the tissue. This cascade is often amplified by the ongoing inflammation, ultimately leading to tissue destruction mediated by multiple cell types [76]. Several processes influence this cascade, in many cases serving to modify the antigenic landscape seen by T cells. These include: (a) inflammatory cytokines in the tissue microenvironment that can upregulate the expression of the enzymes and chemical processes that promote PTMs [35, 75, 77, 78]; (b) inflammation‐induced upregulation of antigen‐processing and presentation pathways including surface MHC expression [79, 80]; (c) exogenous agents such as food antigens and viral infections that may provide a new source of pathogenic epitopes [60, 61]; (d) highly cell‐type‐specific gene programs that may be impossible to replicate within the thymus [81]; (e) MHC/HLA alleles that may exhibit preferences for modified epitopes [69, 70]; and (f) distinct patterns of class I and class II MHC expression, where inflammation can upregulate class II MHC on non‐APCs [2, 3, 4]. In the next section, we will describe citrullination in rheumatoid arthritis (RA) and type 1 diabetes (T1D), formation of Hybrid Insulin Peptides (HIPs) in T1D, and gluten deamidation in celiac disease (CeD) to highlight the immunogenic cascade and its amplifiers (Figure 2). Throughout the review, we will use these three examples to highlight the biology and discovery of PTM‐reactive T cells.

**TABLE 1:** Summary of PTMs shown to be relevant in autoimmune diseases.

PTM	Modification	Autoimmune relevance	References
Citrullination	Arg to Citrulline	RA, T1D, SLE, MS	[35, 36, 37, 38, 39, 40, 41, 42, 43, 44]
Carbamylation	Lys to Homocitrulline	RA	[39, 43, 45, 46, 47]
Acetylation	Lys to Acetyl‐Lys	RA and SLE	[43, 48, 49, 50]
Oxidation	Cys/Met/Trp oxidation	RA	[14, 43, 51, 52, 53]
Phosphorylation	Ser/Thr/Tyr to Phospho‐Se/Thr/Tyr	SLE	[49, 54, 55, 56]
Glycosylation	Lys to Glycosylated‐hydroxy‐lys (Advanced Glycation End products)	T1D, SLE	[57, 58, 59]
Deamidation	Gln to Glu; Asn to Asp	CeD and T1D	[60, 61, 62, 63, 64, 65]
Methylation	Lys to methyl‐Lys or trimethyl‐Lys	SLE	[66]
Hybrid peptides	Peptide‐splicing mediated by Cathepsins	T1D	[65, 67]

**FIGURE 1:**
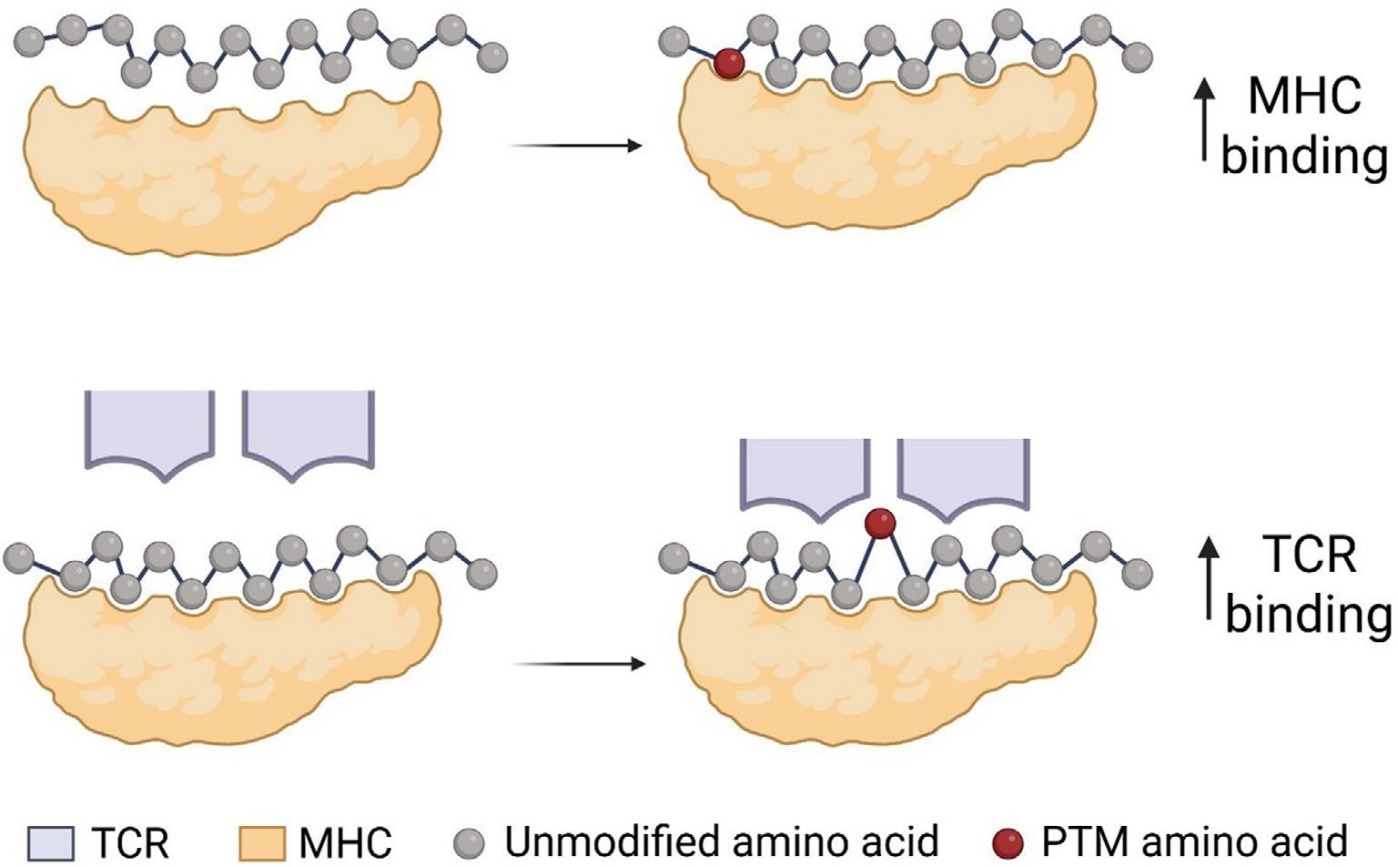
Pathogenic impact of PTMs on MHC/HLA binding (top) and TCR binding (bottom).

**FIGURE 2:**
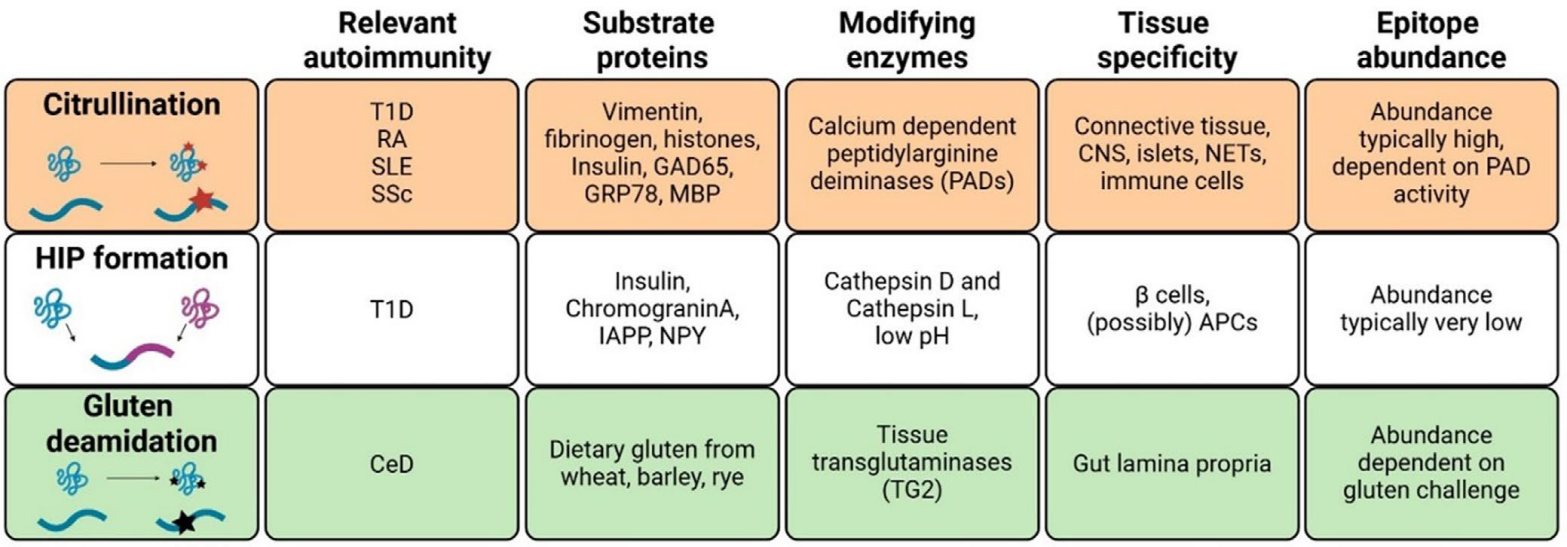
Comparison of the immunobiology of citrullination, HIP formation, and gluten deamidation.

### 1.1 Citrullination

Citrullination is widely regarded as the best‐characterized and most compelling example of an immunogenic PTM in autoimmunity [82]. In RA, anti‐citrullinated protein antibodies (ACPA) serve as highly specific biomarkers and correlate with disease progression and severity [82]. Citrullination itself involves the irreversible conversion of arginine to the non‐canonical amino acid citrulline (R➔Cit), a process mediated by the peptidyl‐arginine deiminase (PAD) enzyme family [83, 84]. There are five PAD enzymes, (PAD1‐4 and 6) which differ in their tissue distribution and expression [85, 86, 87, 88, 89, 90]. Importantly, PAD enzymes are calcium‐dependent and exhibit low basal activity to facilitate essential protein citrullination. However, acute and chronic inflammation can disrupt calcium homeostasis and expose PAD enzymes to abundant and/or atypical protein substrates, resulting in aberrant and widespread protein citrullination [83, 84]. Citrullinated proteins can subsequently be processed into peptides that bind MHC differently or are recognized differently by T cells. Indeed, the strongest RA genetic risk factor is inheritance of certain HLA‐DRB1 alleles (e.g., HLA‐DRB1*04:01, *04:04, 01:01, and 10:01), which encode a conserved amino acid motif at positions 70–74 of the DRB chain [91, 92]. This sequence (QKRAA, QRRAA, or RRRAA), known as the shared epitope, confers a preference within the peptide‐binding groove for neutral citrulline over positively charged arginine. Consequently, citrullinated peptides are likely presented at higher density on APCs, are more stable on MHC/HLA molecules, and are more likely to activate autoreactive CD4+ T cells, which in turn can amplify ACPA responses [93]. Beyond RA, inflammation‐induced formation of citrullinated neoepitopes has been observed across multiple other autoimmune diseases, including multiple sclerosis (e.g., citrullinated myelin basic protein [36, 94]), systemic lupus erythematosus (SLE) (e.g., citrullinated histones induced by NETosis [95, 96, 97]), psoriasis (e.g., citrullinated cathelicidin LL37 [98, 99]) and T1D [37, 100]. Numerous citrullinated epitopes derived from β cell antigens (e.g., insulin, glucose‐regulated protein 78 (GRP78) [35], islet amyloid polypeptide (IAPP), and glutamic acid decarboxylase, 65 kDa isoform (GAD65)) have been shown to elicit immune responses in human T1D and the non‐obese diabetic (NOD) mice [101].

### 1.2 Formation of Hybrid Insulin Peptides

HIPs are a class of PTM neoepitopes formed post‐translationally when peptide fragments of insulin fuse with fragments from other β cell secretory proteins [102]. This process is highly specific to β cells and has been shown to be dependent on cathepsin‐mediated cleavage of β cell proteins, as well as the low pH conditions generated when secretory granules fuse with endolysosomes [103, 104]. As HIPs are not formed in the thymus, T cells reactive to them escape central tolerance. HIP‐reactive T cells are stimulated in pancreatic lymph nodes and induce a pathogenic, pro‐inflammatory phenotype, which promotes autoimmunity [105]. A salient example is found in several diabetogenic CD4+ T cell clones derived from NOD mice, most notably BDC2.5 and BDC6.9 [106], which are sufficient to induce diabetes upon adoptive transfer and were shown to be HIP‐reactive [107]. Since then, HIPs have been established as bona fide driver antigens in T1D [108].

### 1.3 Gluten Deamidation

Similar to HIPs, tissue‐specific post‐translational gluten deamidation has been demonstrated to be pathogenic in CeD [60, 61]. CeD is an autoimmune disorder with significant gastrointestinal pathology caused by intolerance to gluten. Gut pathology is driven by modification of gluten (primarily α‐gliadin from wheat, barley, and rye) by tissue transglutaminase 2 (TG2), where glutamine residues are converted to glutamic acid residues (Q➔E). Deamidation of α‐gliadin peptides leads to presentation of neoepitopes by gut‐associated APCs [60]. Deamidated epitopes are presented particularly well by certain HLA alleles such as HLA‐DQ2.5 and DQ8 [109], which are associated with CeD risk. As these epitopes are not expressed in thymic epithelial cells, T cells recognizing them can escape central tolerance and are stimulated in the periphery upon recognition [110]. CD4+ T cells specific to deamidated gliadin predominantly skew toward a proinflammatory T helper 1 (Th1) phenotype. These pathogenic Th1 cells cause intestinal mucosal damage, promote an inflammatory environment through interferon‐gamma (IFNγ) and interleukin‐15 (IL‐15), which in turn upregulate TG2 [111]. Importantly, this response is heavily dependent on the presence of deamidated gliadin as exemplified by the fact that dietary gluten avoidance is sufficient to prevent CeD flares [110]. This example highlights the key role gluten deamidation plays, along with the endogenous and exogenous factors that amplify its effect.

Together, these examples of key antigenic mechanisms illustrate that PTMs can create neoepitopes, bypass central tolerance, and establish a self‐promoting inflammatory loop with HLA restriction and cytokine feedback acting as key amplifiers of disease. This interplay of environmental and genetic factors serves to amplify the antigenic landscape as well as make it highly dynamic. This also intensifies the challenge of antigen discovery. Traditionally, antigen discovery approaches have been focused on peptides containing the 20 canonical amino acids [112, 113]. Addition of many non‐canonical amino acids, i.e., those carrying PTMs, greatly expands the search space in both size and diversity. This necessitates adapting existing methodologies and inventing new methodologies to interrogate PTM‐specific responses. In the subsequent sections, we will focus on PTM antigen discovery, highlighting four major complementary approaches: (1) proteomics and immunopeptidomics‐based identification of PTM epitopes; (2) computational prediction of MHC binding; (3) identification of antigen‐specific T cell responses using synthetic peptides; and (4) high‐throughput identification of antigens through library‐based screening. For each of these approaches, we will first describe the general principles behind them, followed by specific examples of their application to PTM antigen discovery. We will use citrullination, glutamine deamidation, and HIP formation as examples to highlight these approaches.

## 2 Where's Waldo? Proteomics and Immunopeptidomics

### 2.1 Proteomics

Mass spectrometry (MS)‐based proteomics on enzymatically digested proteins has been a gold standard for detecting PTMs. Typically, proteomics analyses start with a trypsin digest of a given protein milieu, and then the digest is subjected to liquid chromatography (LC) to separate out fragments of different size. Eluted peptides first undergo precise mass measurement of intact peptide ions (MS1 scanning), followed by fragmentation of selected precursor ions to generate sequence‐informative fragment spectra (MS2/3 fragmentation). These mass‐to‐charge (m/z) spectra are then matched computationally against a reference database or searched de novo to assign peptide identity and protein origin. Conventional proteomics can distinguish modified amino acids from their unmodified counterparts based on the mass/charge shifts induced by the modifications. Proteomic profiling of disease‐affected tissues has been instrumental in identifying PTMs. For instance, the detection of HIPs in murine and human β cells and cell lines was key to establishing them as autoantigens in T1D. In a seminal study, Delong et al. performed proteomics on mouse β cell extracts and identified both insulin C‐peptide (InsC) and chromogranin A (ChgA) peptide fragments in antigenic fractions. As these fragments individually were poor at stimulating T cells, unlike the entire fractions, they hypothesized the occurrence of peptide fusions [102]. They generated hypothetical fusion peptides and confirmed that InsC‐ChgA and InsC‐IAPP fusions stimulated diabetogenic CD4+ T cell clones at > 10,000‐fold higher than peptides derived only from InsC, ChgA, or IAPP. Crucially, they were able to demonstrate that two peptide sequences, DLQTLAL‐WSRM (2.5HIP) and DLQTLAL‐NAAR (6.9HIP), were also detectable in the β cell extracts. This was key because of the inherent promiscuity of T cells. T cells have a broad spectrum of cross‐reactivity toward their cognate epitopes, often recognizing mimotopes that are purely synthetic and don't exist physiologically. Showing proteomic evidence for the existence of HIPs was crucial in establishing that they indeed form within β cells. A follow‐up study expanded this by detecting thousands of HIPs in mouse and human β cell extracts [114]. Further proteomic studies by the same group led to the identification of Cathepsin D and Cathepsin L as the primary proteases responsible for generating HIP fragments [104], and showed that HIPs form spontaneously in β cells through an aspartic anhydride intermediate, detailing the mechanistic basis of HIP generation [115]. These results highlight the crucial role of proteomics in confirming the existence of specific PTMs.

Similarly, the ability to detect citrulline by proteomics is crucial to identify specific sites that are citrullinated. For instance, proteomic studies from inflamed synovial joints of RA patients revealed a large set of citrullinated proteins, which were then used to identify T cell responses. This approach, which mapped the ‘citrullinome’ of a target tissue, has been valuable in generating an unbiased view of the PTM landscape [116, 117]. Similar studies to map the ‘acetylome’ and ‘phosphorylome’ have been instrumental in annotating large swathes of PTM sites throughout the mouse and human proteomes. For example, Choudhary et al. used high‐resolution MS to identify 3600 lysine acetylation sites on 1750 proteins, showing that acetylation preferentially targets large macromolecular complexes involved in chromatin remodeling, cell cycle, and splicing, with a regulatory scope rivaling that of phosphorylation [118]. Similarly, a system‐wide proteomic analysis of human cancer cells mapped both acetylation and ubiquitination, identifying over 5000 ubiquitination sites and 1600 acetylation sites, including ~900 previously unannotated lysine modification sites [119]. Furthermore, Olsen et al. used phosphopeptide‐specific MS‐based sequencing to map phosphorylation sites as a function of stimulus, time, and subcellular location, cataloguing 6600 phosphorylation sites across 2244 proteins in Epidermal Growth Factor‐stimulated HeLa cells [120]. Together, these proteomic efforts illustrate how systematic PTM mapping has expanded annotated acetylation and phosphorylation sites from a handful of well‐studied examples to many thousands of sites across the proteome, establishing the technical and conceptual precedent for similarly comprehensive site‐level mapping of other PTMs.

Identifying PTMs by mass spectrometry faces two main challenges. First, many biologically distinct modifications produce nearly identical mass shifts. For example, deamidation and citrullination both induce an identical +0.984 Da shift [121]; whereas trimethylation (+42.047 Da) is very similar to acetylation (+42.011 Da). By contrast, PTMs like phosphorylation (+79.966 Da) or ubiquitination (+114.043 Da) produce large, easily resolved mass shifts. This necessitates high‐resolution and high‐mass‐accuracy MS instruments as well as novel approaches for spectral deconvolution. Without this technical prowess, near‐isobaric modifications cannot be reliably told apart, which is especially problematic for histones where trimethylation and acetylation frequently compete for the same lysine residues [122, 123]. Second, repetitive sequences make it hard to assign a PTM to the correct residue or region. For example, in CeD, the 33‐mer immunodominant α‐gliadin peptide (LQLQPFPQPQLPYPQPQLPYPQPQLPYPQPQPF) contains six overlapping repeats of near‐identical proline/glutamine‐rich motifs. If this peptide is deamidated, a fragment ion often cannot distinguish which of the repeated glutamine residues carries the modification, since multiple positions produce the same mass shift on backbones that look almost identical to the search algorithm [124].

Together, these examples show that conventional proteomics has worked well for cataloguing individual PTMs and their autoantigens. A key missing piece from proteomics is that it doesn't resolve which PTM‐bearing epitopes are presented on MHC. Immunopeptidomics directly addresses this by surveying the peptides actually being presented on MHC molecules, using new computational tools and modification‐aware search strategies to discover which PTMs are immunologically relevant in an unbiased way.

### 2.2 Immunopeptidomics

Immunopeptidomics is essentially a narrow and focused proteomics search of MHC‐bound peptides [125]. Because MHC/HLA molecules bind peptides non‐covalently rather than through a permanent chemical bond, this collection of bound peptides, called the ‘immunopeptidome’, can be extracted biochemically. This typically involves isolation of pMHC complexes using immunoprecipitation with anti‐MHC antibodies. The immune complexes are then treated with mild acid to release the peptides from MHC. Another key difference from traditional proteomics is that there is no trypsin digestion used during peptide elution, which preserves the physiological state of the epitopes prior to MS. Analyses of immunopeptidomics datasets are largely performed using similar pipelines as proteomics, with some nuanced adjustments that will be discussed in the next section. Immunopeptidomics analyses performed on class I and II MHC have been instrumental in profiling PTMs across many different contexts [125, 126]. In our experience, virtually any immunopeptidomics experiment will detect PTM‐containing epitopes, depending on the data acquisition and analysis settings. For instance, our studies profiling I‐Ek‐restricted epitopes in lupus‐prone MRL/lpr mice identified 1382 peptides derived from 493 source proteins. A PTM‐focused search showed 82 citrullinated peptides from 59 proteins [127]. Oxidation or N‐terminal acetylation were also found in ~210 peptides; however, these can often be sample preparation artifacts. Importantly, immunopeptidomics allows us to deconvolute peptides at an MHC/HLA allele level by using allele‐specific antibodies, increasing the resolution of analyses. This ability uniquely allows us to profile which PTMs are amenable to presentation across different MHC alleles, or across species. For instance, independent immunopeptidomics analyses on pancreatic islets from NOD mice or from T1D donors showed numerous conserved source proteins [128, 129]. Importantly, several PTMs such as citrullination and deamidation were also identified across both datasets, highlighting that such analyses are able to profile PTM‐containing epitopes at high resolution. A compilation of immunopeptidomics from cancer cell lines identified 372,720 cancer‐associated PTM‐containing peptides [130]. A deeper review of immunopeptidomics for autoimmunity with disease‐specific HLA associations and datasets has been performed by us and other groups [125, 131], and hence we will focus on how immunopeptidomics can inform antigen discovery of PTM epitopes and what its limitations are.

### 2.3 Data Analysis Pipelines for PTM Search

Peptide‐level MS data can be collected using one of two acquisition strategies: data‐dependent acquisition (DDA) or data‐independent acquisition (DIA). DDA, in which the instrument selects and fragments the most abundant precursor ions, has long been the standard for navigating the complexity of database searches without enzyme‐specific cleavage constraints. However, DIA, which fragments all precursors within defined m/z windows independent of their abundance, is being increasingly adopted in immunopeptidomics workflows [132, 133], because DIA more thoroughly samples the m/z scan space than DDA. Therefore, it can improve sensitivity and reproducibility, particularly for low‐abundance HLA‐bound peptides, although its performance remains heavily dependent on the quality and coverage of the reference spectral library used for matching. Despite the availability of several DIA data‐processing tools, the immunopeptidomics community has not converged on a consensus pipeline for accurate and in‐depth HLA‐peptide identification. A recent benchmarking study directly compared four of the most widely used library‐based DIA pipelines including Skyline, Spectronaut, DIA‐NN, and PEAKS, highlighting substantial differences in performance and underscoring the need for continued methodological standardization in this field [133, 134, 135, 136, 137].

Once acquired, spectra are matched to peptide sequences through database searching. Until recently, closed searches, in which a predefined list of expected modifications and their corresponding mass shifts is specified in advance, represented the standard approach to peptide identification in immunopeptidomics, mirroring conventional strategies used in shotgun proteomics [138, 139]. While effective when the relevant PTMs are known a priori, this approach is inherently limited by the problem that any modification not included in the search parameters will go undetected. Because the combinatorial search space encompassing all potential peptides and their possible modifications is prohibitively large, conventional closed‐search strategies constrain the range of PTMs that can be considered, resulting in a substantial proportion of unassigned spectra. This limitation has driven a major methodological shift in the field toward open (mass‐tolerant) searching, which expands the effective search space and reduces the number of unexplained spectra by identifying peptides bearing mass shifts consistent with known or unanticipated modifications, as well as sequence variants [140, 141, 142]. Open search is therefore particularly well suited to exploring uncharted immunopeptidomes, for example, in the discovery of non‐canonical mutations or viral and bacterial antigens and to studies in which non‐enzymatic cleavage, unexpected PTMs, or the absence of a pre‐existing sample‐specific spectral library preclude reliable identification through closed‐search or conventional library‐based DIA approaches [143, 144].

Despite the directness and power of immunopeptidomics to profile PTM epitopes, there are several limitations associated with it, which restrict its wide use. A key limitation of immunopeptidomics is that it is likely profiling < 1% of the actual immunopeptidome. Typical experiments yield hundreds to thousands of peptides per sample, with only tens to hundreds being detected with PTMs. Given that the number of MHC molecules per cell is estimated to be > 10^5^, a majority of presented peptides are likely missed by immunopeptidomics. This is despite the need for a large amount of starting material. In our experience, experiments with 50–100 million APCs yield 1000–2000 peptides. This suggests that immunopeptidomics is able to detect only a small fraction of presented peptides. Peptide abundance and affinity for MHC are the two most likely factors that dictate whether they are detected [145]. In many cases, obtaining sufficiently high numbers of disease‐relevant tissue or APCs may not be possible or feasible, precluding being able to perform immunopeptidomics. For instance, in T1D, obtaining sufficient β cells from cadaveric donors is challenging, limiting analyses to healthy islets subjected to in vitro stimulation or to β cell lines. Another limitation worth mentioning is that both proteomic PTM profiling and immunopeptidomics are, by nature, static snapshots, meaning a single MS run reports the modification and presentation landscape at one moment, in one tissue sample, under one set of conditions. But MHC presentation itself is highly dynamic, the peptide repertoire displayed on a cell surface turns over continuously and shifts with the cell's activation state, microenvironment, and metabolic state. Moreover, the mechanisms that lead to PTMs are often inflammation‐driven rather than constitutive. This is a key experimental limitation, necessitating optimization of a strategy to capture relevant antigens while avoiding ‘searching under the street light’ scenario. Moreover, this is also a limitation for causal inference, where immunopeptidomics can establish that a PTM‐containing peptide is being presented, but it has virtually no information on the time at which and the conditions under which it was generated. The static nature of immunopeptidomics also disallows any inference of dynamics of a given PTM, which can only be learnt through taking multiple static snapshots across a timeline.

Despite these limitations, immunopeptidomics can be highly informative, leading to identification of PTM‐containing epitopes that can directly feed into T cell antigen discovery. Complementary approaches such as EPI‐SCAN [146], can also be used to infer MHC‐bound peptides by combining library‐scale expression of genetically defined pMHC molecules with high‐throughput sequencing. However, as these are fairly specialized techniques, they are not accessible widely. For something that is widely accessible, we now turn to computational predictions of MHC‐bound epitopes.

## 3 The Sorting Hat—MHC Binding Predictions

Several computational tools have been developed to predict peptide–MHC binding across both human and mouse alleles. MHC binding prediction tools estimate how well a given peptide will bind to a specific MHC allele, typically outputting a predicted binding affinity or a percentile rank score. NetMHCpan‐4.1 and NetMHCIIpan‐4.0 are among the most widely used pan‐specific predictors for class I and class II binding, respectively, while MHCflurry is an open‐source, scriptable alternative with an added antigen‐processing component [147, 148, 149, 150]. Other tools such as MixMHCpred and MixMHC2pred use motif‐deconvolution strategies particularly suited to mass‐spectrometry data [151], whereas MHCSeqNet2 focuses specifically on improving predictions for alleles with limited data [152]. Massive datasets of MHC‐bound peptides, often those generated by immunopeptidomics and/or reported on the Immune Epitope Database (IEDB) with experimental evidence, are used as training data. IEDB [153], which serves as a central repository and resource for MHC‐bound peptides, has > 1 million MHC‐bound peptides as of June 2026. Out of these peptides, ~46,000 contain one of the following modifications: acetylation, citrullination, cysteinylation, deamidation, methylation, nitration, or phosphorylation, exemplifying the scale of datasets available for learning MHC binding prediction.

The early prediction tools used position‐specific scoring matrices to identify positional preferences for amino acid binding for a given MHC allele [154, 155, 156, 157]. Newer tools mostly rely on neural networks to model MHC binding. The core biological logic being learned is the anchor residue pattern [158]: each MHC allele has a peptide‐binding groove containing pockets with preferences for particular residues. Typically positions 2 and 9 of 9‐mer epitopes are anchor residues for class I MHC alleles. Class II alleles are harder to predict due to the open‐ended nature of the peptide‐binding groove; however, most tools often predict core 9‐mer motifs. Prediction algorithms essentially learn allele‐specific sequence motifs and score new peptides by how well they fit while also capturing more subtle, non‐anchor position effects that simple motif‐matching would miss. For MHC class II prediction, the problem is harder because the peptide‐binding groove is open‐ended rather than closed—so the tool also has to predict which 9‐residue “core” segment within a longer peptide actually sits in the groove since class II peptides are typically 13–25 residues with flanking overhangs outside the binding pocket, but the fundamental scoring approach is similar.

MHC binding predictions are highly valuable, as they help identify potentially immunogenic epitopes as a way of prioritizing peptides to be used to detect T cell responses. The accuracy of these algorithms is generally strong for common, well‐characterized alleles, and is better for class I MHC than class II. The prediction quality scales directly with how much training data exists for a given allele but it drops noticeably for rare, less‐studied, or non‐human alleles that are underrepresented in these datasets [159, 160]. When applied to immunopeptidomics data specifically, tools trained on eluted‐ligand data rather than affinity data alone tend to be more useful, since they better reflect naturally processed and presented peptides rather than just theoretical binding potential. This makes them helpful for tasks like deconvoluting multi‐allelic mass‐spec datasets, assigning peptides to likely alleles, and validating which peptides identified by MS are genuinely presented rather than being background contaminants. Recently, we developed an interaction language model (iLM), called Sliding Window INteraction Grammar (SWING) as a tool to learn pMHC binding biology. Rather than learning allele‐specific patterns the way NetMHCpan or MHCflurry do, SWING learns a more generalized representation of the pMHC interactions itself, which allows it to maintain reasonable predictive accuracy even for alleles it has never seen, including alleles that are functionally distant from its training dataset. It also generalizes across MHC classes and species in ways existing tools are not built to do. Indeed, we were able to show that iLMs trained on human class I and II alleles were able to accurately predict peptides derived from murine immunopeptidomics datasets generated by Wan et al., as well as our own dataset from lupus‐prone mice [126, 127].

A key limitation of MHC binding prediction algorithms is that they are typically limited to the canonical amino acids. They can certainly be used for HIPs and deamidation, which generate epitopes only with canonical sequences. However, for PTMs such as citrullination, they cannot be used. Early studies on citrullinated epitopes led to algorithms that used positional preferences for predicting citrullinated epitopes on HLA‐DR [161]. But there are no general tools for predicting the effect of citrullination on MHC binding. To our knowledge, the only algorithms that can predict PTM epitopes focus on phosphorylation. These algorithms used immunopeptidomics data containing phosphorylated epitopes and used the NNAlign framework to predict MHC binding. PhosMHCpred and NetMHCphosPan [162] were developed for class I MHC binding predictions and revealed a strong preference for the phosphorylated residue at position 4 of the binding motif, with enrichment of proline at position 5 and arginine at position 1. The same group developed NetMHCIIphosPan for class II MHC binding predictions and confirmed the exquisite specificity of T cells in distinguishing phosphorylated/unmodified epitopes. While these tools represent a great advance in being able to predict MHC binding of PTM epitopes, they are sorely needed for PTMs beyond phosphorylation [163].

Taken together, proteomics and immunopeptidomics coupled with MHC binding predictions enable one to identify potentially immunogenic peptides. The next step is to usually test for T cell responses that recognize them. This is done through two complementary approaches: antigen‐directed approaches that start with candidate peptides and TCR‐directed approaches that use large‐scale libraries. In the subsequent sections, we will cover the use of these approaches to identifying PTM‐reactive T cells.

## 4 The Bat Signal—Antigen‐Directed Discovery

Antigen directed approaches ask whether given a set of candidate antigens, can one identify T cells reactive to them. Starting with a small set of candidate peptides, these approaches rely on a readout of T cell binding and/or recognition. The advantages offered by these approaches include: high sensitivity of detection down to as few as one epitope‐specific cell per million T cells; relatively straightforward use without needing highly specialized techniques; providing a quantitative readout of immune responses; and the ability to interrogate large polyclonal repertoires [164, 165]. Two key antigen‐directed assays include: pMHC multimers and functional assays. We will discuss both these approaches as follows:

### 4.1 Soluble pMHC Multimer Reagents

Soluble pMHC complexes containing desired peptides can be generated and labeled with fluorophores and multimerized [166]. Fluorescently labeled pMHC multimers are used to stain T cells for flow cytometry‐based detection. Staining with pMHC multimers is typically highly sensitive, being able to detect frequencies of antigen‐specific T cells as low as 0.01%. Furthermore, pMHC multimers allow sorting of antigen‐specific T cells, allowing for in‐depth investigations with single‐cell RNA sequencing or other single‐cell assays. Co‐staining with surface or intracellular markers can further aid in phenotyping of antigen‐specific cells. Specifically, pMHC tetramers have been used extensively to profile immune responses across many different disease contexts, including profiling of PTM‐specific T cell responses in autoimmunity.

Citrullination, for example, has been extensively studied in RA and more recently T1D using pMHC tetramers. Using a panel of citrullinated epitopes derived from vimentin, cartilage intermediate‐layer protein (CILP), filaggrin, α‐enolase, and fibrinogen, James et al. [167] profiled Th1 cells in HLA‐DRB1*04:01 transgenic mice and RA patients. Furthermore, they showed that RA patients showed a higher propensity of citrulline‐reactive T cells toward a Th1‐skewed, antigen‐experienced phenotype. A similar study demonstrated the presence of citrulline‐reactive CD4+ T cells in RA patients [168] and people at risk for RA. Interestingly, a similar panel was used to suggest that citrulline‐reactive T cells generated following immunization showed a skew toward a more regulatory phenotype, suggesting that there is peripheral tolerance toward these epitopes [72, 169].

Detection of gluten/gliadin‐reactive T cells with pMHC tetramers has been well‐established for almost two decades. Raki et al. used HLA‐DQ2‐gliadin tetramers to show that gluten challenge induces gut‐homing T cells in CeD patients [170]. Using HLA‐DQ2.5‐tetramers presenting two closely related epitopes (glia‐α1a (PFPQPELPY) and glia‐ω1 (PFPQPEQPF)), Dahal‐Koirala et al. showed that TCRs from CeD patients show high specificity toward one of the two epitopes with minimal cross‐reactivity [171, 172].

Similarly, in T1D, using pMHC tetramers presenting HIPs has been useful to interrogate pathogenic T cell responses in NOD mice and humans. In NOD mice, pMHC tetramers presenting Ins‐ChgA (2.5HIP) and Ins‐IAPP (6.9HIP) were used to profile HIP‐reactive islet infiltrating T cells and showed increasing frequencies with disease progression [102, 105, 107, 114, 173]. HLA‐DQ8 tetramers presenting HIPs were also used to profile T cells from T1D patients [105]. A recent study showed that T cells reactive to the 2.5HIP and 6.9HIP exhibited an inflammatory skew compared to a regulatory skew shown by unmodified insulin B (InsB)‐reactive T cells [174]. These studies highlight the key advantages offered by pMHC multimer reagents, which can, at least in principle, be generated from any PTM epitope and any MHC allele [173, 174]. These reagents are generated by expressing soluble MHC molecules in *E. coli* and loading them with synthetic peptides. The stability of soluble pMHC is dependent on the affinity of the peptide to MHC. Therefore, any given synthetic peptide can be used to generate pMHC multimers, given sufficient binding affinity. While sensitive, pMHC multimers are limited by very low throughput of interrogating epitopes (often < 10), complex and laborious technical protocols for generating tetramers, and high level of instability of class II tetramer reagents [166]. This is especially important, as many PTMs in autoimmunity are likely class II MHC‐restricted. Approaches such as DNA‐barcoded tetramers [175, 176] or combinatorial fluorophore labeling [177] can increase the throughput, but such approaches have not been deployed for PTM epitopes yet. In particular, the instability of class II tetramers necessitates that only high‐affinity peptides can be used to make these reagents. Due to these limitations, pMHC multimers are often best suited when there is a high level of confidence in the peptides to be tested.

### 4.2 Functional Assays

Functional assays rely on high sensitivity and specificity of T cells to their cognate epitopes as well as rapid upregulation of surface markers and cytokines upon their recognition on APCs. In these assays, polyclonal T cell repertoires are stimulated with individual synthetic peptides or pools of peptides. After a short incubation, functional responses from T cells responding to the peptides are measured colorimetrically or using flow cytometry. Functional responses can be measured in an aggregate manner in Enzyme‐linked Immunosorbent Assays (ELISAs) or cell proliferation, or on a per‐cell basis with Enzyme‐Linked ImmunoSpot (ELISPOTs), intracellular cytokine staining, or activation‐induced marker (AIM) assays. Production of IFNγ, Interleukin‐10 (IL‐10), and Interleukin‐2 (IL‐2), or cell proliferation with radioisotope incorporation has been classically used to detect antigen‐specific responses [112]. These readouts are often straightforward and inexpensive to use, and allow processing of many samples at once. They also have the ability for coarse testing of reactivity to up to hundreds of peptides at once, e.g., when using peptide megapools. For instance, Hohenstein et al. used a library of 240 synthetic Insulin C HIP peptides to identify novel reactivities in T1D patients [178]. They are also versatile, as they can be used either for polyclonal T cell populations or individual T cell clones or lines. There are numerous examples of these assays being used to detect responses to PTM epitopes [38, 179, 180]. A key limitation of the aggregate measurements is a lack of quantification of frequency of antigen‐specific T cells. When these assays are performed with peptide pools, they also are limited by needing to deconvolute fine specificity, which may or may not be possible depending on sample availability. Per‐cell measurements by ELISPOTs and AIM assays can also be performed with individual or pooled peptides. ELISPOTs provide a sensitive way of colorimetric quantification of T cells that produce specific cytokines (often IFNγ, IL‐10, or IL‐2). ELISPOTs are often used as ‘gold‐standard’ assays for measuring and quantifying antigen‐specific responses. Intracellular cytokine staining and surface staining for activation markers such as CD69, CD25, OX‐40, and others can also provide a sensitive way to measure antigen‐specific responses. These assays can provide an understanding of the cellular states and phenotypes within the antigen‐specific population, unlike aggregate assays. Importantly, these assays allow detection and quantification of as few as one antigen‐specific cell per million. Naturally, functional assays are often the first line of assays used to measure reactivity to PTM epitopes. T cell responses to citrullinated epitopes in RA and T1D, HIPs in T1D, and deamidated gliadin have been studied using functional assays [67, 181, 182]. Despite their wide use, there are a few limitations of these assays. The scale at which these can be performed is still limited to hundreds, as they rely on synthetic peptides. Depending on the tissue and antigen, functional readouts may not reflect true quantification, especially if antigen‐specific cells are functionally hyporesponsive due to exhaustion or anergy. Importantly, most functional assays provide limited biological insights beyond quantitative detection and coarse phenotyping. An exception is AIM assays, where cells expressing early activation markers can be phenotyped finely using large panels, or sorted with fluorescence‐assisted cell sorting (FACS) and sequenced to assess TCR repertoires. Another limitation is that functional assays can suffer from variability across sites, flow cytometers, operators, and technical replicates. Despite these, functional assays are the first‐line choice for detecting PTM‐specific T cells.

## 5 The Hunger Games—TCR‐Directed Discovery

Unlike antigen‐directed approaches described above, TCR‐directed approaches often do not need a priori information of candidate antigens. TCR‐directed approaches ask whether, given a T cell clone or a TCR of interest, one can identify its cognate epitopes. These methods screen a large number of epitopes against a single or a small number of TCRs. Epitope libraries can range from small, bespoke libraries consisting of hundreds of epitopes to fully degenerate libraries consisting of billions of epitopes. The libraries are screened against a TCR of interest, and epitopes that either bind to the TCR or can stimulate a functional response downstream of the TCR are positively selected. Through one or more rounds of positive selection, cognate epitopes recognized by the TCRs are enriched and identified. Two methods allow screening of fully degenerate libraries: combinatorial peptide libraries and yeast display, whereas cell‐based libraries are more customizable.

### 5.1 Combinatorial Peptide Libraries

A positional scanning synthetic combinatorial library (PS‐SCL) [183] is built as a set of peptide mixtures in which one position along the peptide is held fixed to a single amino acid, while all other positions contain an equimolar mixture of many/all amino acids. This setup is repeated so every position in an epitope gets its own “fixed” scan across the full panel of amino acids. By testing a T cell or TCR against each mixture and seeing which fixed residues at which positions drive the strongest response, a consensus recognition motif can be inferred. This allows for identification of epitopes that strongly stimulate the TCR [183]. A major advantage of PS‐SCLs is that no a priori knowledge of the antigen is needed, allowing one to explore the entire search space. A major caveat is that PS‐SCLs often identify mimotopes—epitopes that stimulate the TCR but are not actual physiological epitopes, and in some cases may bear little resemblance to the actual epitope. A key example of PS‐SCLs was their use on the BDC2.5 TCR, which led to identification of several mimotopes [184] including the p31 mimotope (YVRPLWVRME), which is still used as a gold standard peptide for activating BDC2.5 T cells. While this epitope did not match any known sequence, it did reveal the WXRM(D/E) motif, which was later found in the physiological HIPs that were identified to be targeted by BDC2.5 [102]. This example highlights both the strengths and weaknesses of PS‐SCLs. PS‐SCLs are almost always successful at identifying peptides that can stimulate the TCR under investigation; however, there is a high likelihood of identifying only mimotopes that fail to map to actual epitopes, as they start with a completely degenerate set of peptides. They can often be used to determine MHC anchor residues, which can be helpful in narrowing down the peptide search space [185, 186, 187, 188]. PS‐SCLs have been used to deconvolute TCRs in autoimmunity including the example described above [185]. The use of PS‐SCLs for PTMs has been limited. For PTMs that can be encoded by the canonical amino acids, such as HIPs and glutamine deamidation, PS‐SCLs can be useful, but no direct examples exist. While SCLs containing phosphorylated amino acids have been used to identify kinase substrates, they have not been used for T cell epitope mapping [189]. A major caveat of PS‐SCLs is that they are extremely laborious and expensive, and have more recently fallen out of favor, with newer and superior methods like yeast display being preferred.

### 5.2 Yeast Display

Yeast display works by engineering yeast cells to express a library of pMHC complexes on their surface, which contain peptides covalently linked to MHC, thereby allowing genetic encoding [190]. Large genetically encoded libraries that are either fully degenerate or have positional biases are expressed on yeast. This yeast library is then incubated with a soluble, fluorescently labeled version of the TCR of interest. Yeast with bound TCRs are FACS‐sorted and allowed to grow. Over several rounds of sorting and regrowth, a library containing billions of epitopes is narrowed down to tens to hundreds of epitopes. The peptide‐encoding DNA from the sorted yeast is sequenced to reveal candidate epitopes recognized by the TCR. Yeast display has been used to deconvolute TCRs from TILs and autoimmunity [190, 191]. A salient example of this is the deconvolution of the specificity of two HLA‐DR15‐restricted TCRs from an MS patient [190]. Yeast display also tends to identify mimotopes rather than actual epitopes. The mapping‐back problem is easier for yeast display, as it identifies actual epitope sequences and not just positional preferences, therefore allowing true mapping‐back of mimotopes to physiological sequences [190]. Key limitations of yeast display include the requirement for engineering individual MHC alleles to enhance stability and for producing soluble TCRs, which are both non‐trivial and laborious. To our knowledge, yeast display has not been adopted to investigating PTMs, but similar to PS‐SCLs, HIPs and deamidated peptides can likely be identified through it.

### 5.3 Cell‐Based Libraries

The next set of methods uses designer libraries expressed in reporter cells for TCR‐directed antigen discovery. The Elledge laboratory developed the TScan and TScan‐II platforms, which use target cells that are engineered to express a genome‐wide library of ORF‐derived peptide fragments [192, 193]. The peptide fragments are processed and presented on MHC via the endogenous machinery. Target cells are co‐cultured with the TCR‐expressing primary T cells, which recognize their cognate epitope and release granzyme B. The target cells use a fluorescent reporter that is activated by granzyme B, allowing identification of cells that are recognized by the TCR under investigation. Sequencing of reporter enriched libraries allows for identification of peptides recognized by the TCR. These platforms can be used effectively for antigen discovery, but as they rely on genetically encoded libraries, their use for PTMs has not been demonstrated. Another set of methods uses pMHC complexes that consist of peptides genetically and covalently linked to MHC. These receptors can be expressed on the cell surface and used to present a desired set of peptides in a library format. Recognition of peptides can be read out either through trogocytosis‐mediated cell–cell label transfer to APCs [194], or by stimulation of TCR‐expressing T cells [195]. Alternatively, pMHC complexes fused to a signaling domain can be used to directly report recognition. Using these tools, libraries of desired pMHC complexes are constructed and screened against TCRs of interest. As these are genetically encoded, they are inherently limited to incorporating PTMs with canonical amino acids. To our knowledge, our group has the only published report that uses cell‐based libraries for PTM discovery.

Our laboratory developed Signaling and Antigen‐presenting Bifunctional Receptors, which fuse pMHC complexes with CD28‐CD3ζ signaling domains [165]. SABRs presenting desired peptides covalently linked to MHC can be expressed in NFAT‐GFP‐Jurkat reporter cells. Recognition of a SABR by its cognate TCR triggers a signal through the CD28‐CD3ζ domains, leading to rapid expression of GFP. We showed that SABRs presenting the 2.5HIP were able to induce GFP upon recognition of Jurkat cells expressing the BDC2.5 TCR. Using an immunopeptidomics dataset that eluted I‐Ag7‐bound peptides from NOD mice, we built a SABR library of 4075 epitopes, which contained several known HIPs. We showed that screening BDC2.5 TCR against this library successfully identified its cognate epitopes including the above‐mentioned p31 mimotope as well as 2.5HIP and its related epitopes. We then used this library to de‐orphanize 10 out of the top 35 clonally expanded TCRs from islet‐infiltrating CD4+ T cells from NOD mice. We showed that 6 out of these 10 TCRs recognized InsC‐ChgA (2.5HIP equivalent) or InsC‐IAPP (6.9HIP equivalent) HIPs. Given the preponderance of these TCRs that constituted ~10% of the clonally expanded repertoire, we hypothesized that more HIP reactivities exist within the repertoire. As the foundation for a HIP‐focused screen, we used a proteomic dataset, in which Cathepsin cleavage products of β cell proteins were profiled and assembled into a putative in silico library of HIPs. We built a library of 2561 putative HIPs that consisted of left‐half fragments from InsC and right‐half fragments from the Wiles et al. 2019 dataset [114]. We screened this library against a TCR that failed previous de‐orphanization efforts and identified several HIPs recognized by it. To our knowledge, this was the first high‐throughput HIP‐focused antigen discovery approach. Importantly, the existence of immunopeptidomics and proteomics datasets was instrumental to the design of this focused library. Similar efforts on InsB chain HIPs and human HIPs are underway using this approach. Other PTMs such as asparagine deamidation [196], and recently described cysteine to serine conversion may also be amenable to SABR libraries and related approaches.

As alluded to, while TCR‐directed approaches offer several advantages over antigen‐directed approaches, they are significantly limited in the capacity to profile PTMs beyond the canonical amino acids. Sporadic efforts to couple these approaches to PTMs have been reported, but not for T cell antigen discovery. For instance, a phage display approach called Phage ImmunoPrecipitation Sequencing (PhIP‐Seq), which was developed for B cell antigen discovery, was adapted to interrogate citrullinated targets of ACPAs [197]. PhIP‐Seq works by displaying a highly diverse library of peptides on the surface of bacteriophage. Peptide libraries are typically constructed based on proteome‐wide tiles akin to T‐scan, and are subjected to selection akin to yeast display using antibodies or patient serum. The Roman‐Melendez et al. study built a phage‐displayed peptidome, and modified it using PAD enzyme treatment to convert arginine residues to citrulline [197]. Using PAD‐modified PhIP‐Seq, they identified novel targets of ACPAs and profiled the human citrullinome. Another approach is to encode for residues that mimic PTMs. In library‐scale approaches, phosphorylated serine, tyrosine, and threonine can be replaced by aspartate or glutamate, which mimic the negative charge of the phosphate group. These phosphomimetic mutations can be incorporated into proteins, allowing a profiling of the biochemical effects of phosphorylation. The application of these mutations to antigen discovery has been limited. Interestingly, a report showed that a leukemia phospho‐neoantigen, pMLL747–755, was recognized by a TCR called TCR27 [198]. They found that replacing the pSer at position P4 with either serine or phosphomimetic aspartate/glutamate did not affect the peptide–MHC conformation or binding affinity, but abrogated TCR27‐dependent T cell activation and weakened binding between TCR27 and the pMHC complex. This highlights that phosphomimetic amino acids can be used to profile MHC binding, but may have limitations in identifying TCR binding. These examples demonstrate that genetically encoded TCR‐directed methods can be adapted for PTMs, and highlight the potential for such methods.

In the next section, we will compare three PTMs—citrullination, HIP formation, and glutamine deamidation. For each PTM, we will focus on how its biology and immunogenicity affect the approaches used for discovery. We will draw parallels in the historical discovery of T cell responses to these PTMs and highlight the distinct challenges faced in their discovery from biological and technical considerations.

## 6 The Three Amigos—Citrullination, HIP Formation, and Glutamine Deamidation

Citrullination, as described above, has been observed in many autoimmune diseases including RA, T1D, and SLE [199, 200, 201]. HIP formation, on the other hand, is found exclusively in T1D [105]. Gluten deamidation is associated with CeD but is highly dependent on the presence of dietary gluten [202]. Below, we compare how their biology is distinct when viewed from an antigen‐discovery standpoint, and how amenable these are for the various discovery approaches that were described above (Figure 3).

**FIGURE 3:**
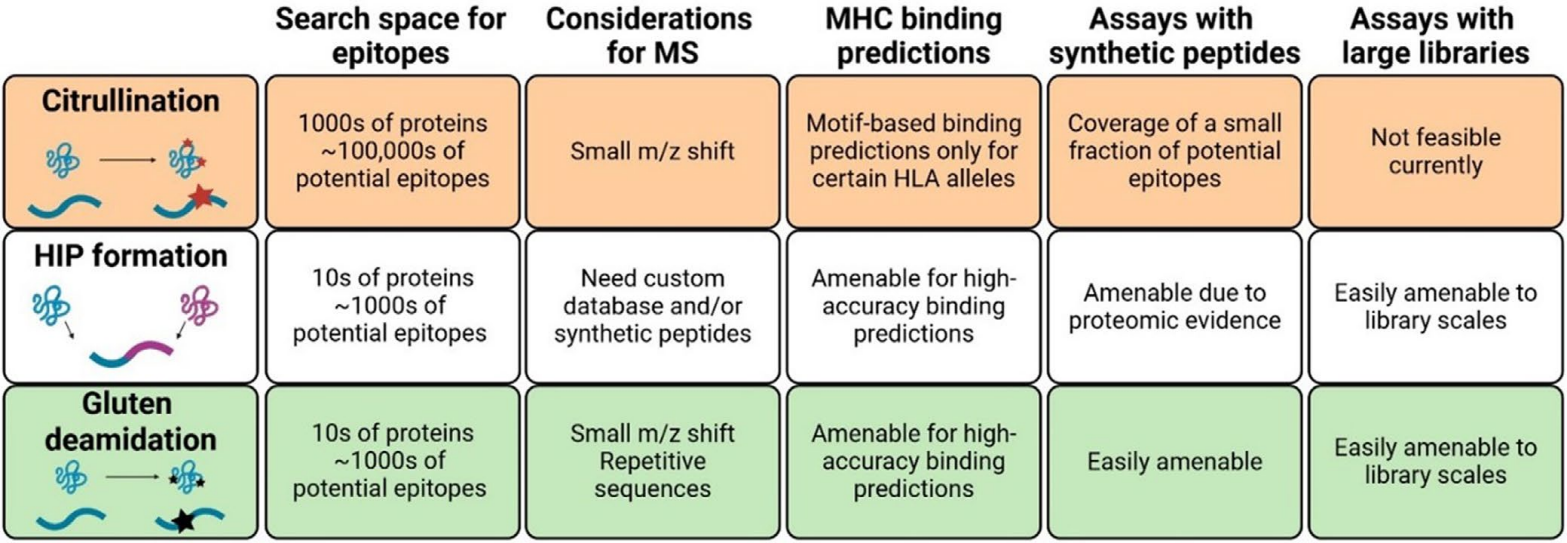
Comparison of citrullination, HIP formation, and gluten deamidation from the perspective of antigen discovery.

### 6.1 Substrates and Tissue Specificity

Citrullination is mediated by PAD enzymes, which show broad expression patterns across tissues [203]. Notably, PADs activity is upregulated in inflamed synovium in RA, in neutrophils undergoing NETosis at sites of inflammation, and in various epithelial and immune cells under oxidative or inflammatory stress [204, 205]. Therefore, citrullination is found across a large number of proteins with broad tissue distribution. Proteomic analyses have revealed ~3245 proteins with experimentally determined citrullination sites across the mouse proteome, resulting in > 30,000 possible citrullinated epitopes [206, 207]. This creates a formidable challenge, where approaches that use synthetic peptides can tackle a very small fraction of the potential epitopes. On the other hand, HIPs are far more tissue‐restricted, as in the case of T1D they form specifically inside pancreatic β‐cell secretory granules. Proteomic analyses done by Wiles et al. showed thousands of putative HIPs [114]. This is well within the range of TCR‐directed methods. In comparison, gliadin deamidation is driven by TG2, which is upregulated predominantly in the small intestinal lamina propria during inflammation, and gut permeability allows partially digested gliadin peptides to encounter the enzyme [208, 209]. Our own analysis of gliadin sequences from wheat, barley, and rye with potential deamidation sites leads to ~5000 potential epitopes, which is also well within the range of TCR‐directed approaches. For both HIPs and deamidated gliadin, MHC binding predictions can narrow potential epitopes down to hundreds, which is reasonable for synthetic peptide‐based approaches. Therefore, HIPs and deamidated gliadins are amenable for genetically encoded approaches as well as synthetic peptides. In contrast, study of citrullination is limited to synthetic peptides, as it cannot be genetically encoded. Therefore, the ability to interrogate citrullinated epitopes is typically in hundreds of peptides, which covers only a small fraction of potential epitopes, highlighting the need for novel, high‐throughput approaches.

### 6.2 Distribution and Accessibility of T Cell Responses Recognizing PTMs

The key determinant of successful identification of PTM‐reactive T cells is their frequency in the tissue to be investigated and the quality and magnitude of their response. It is well established that pathogenic T cells in autoimmunity are clonally expanded within the affected tissue, which is a useful way to identify relevant T cells [165, 210, 211]. If clonally expanded T cells can be identified through single‐cell RNA sequencing of the affected tissues, they can be amenable to TCR‐directed antigen discovery [165]. Citrullination in RA has been predominantly studied in synovial joints [199], whereas in T1D, it is thought to occur in islets and pancreatic lymph nodes [200]. Sampling of synovial joints from RA patients is feasible and has been widely demonstrated [212], whereas sampling of pancreata or pLNs from living donors is virtually impossible. HIP formation has largely been demonstrated to occur in pancreata and pLNs in T1D [105], thereby limiting direct access to pathogenic T cells from living donors. Gliadin deamidation occurs primarily in the gut, which is accessible through gut biopsies [182]. Therefore, simply due to the affected tissue, pathogenic HIP‐reactive T cells are considerably challenging to sample from living donors, whereas cit‐reactive and deamidated gliadin‐reactive T cells can be profiled with various levels of difficulty depending on the context. Mouse‐to‐human overlap is also a significant factor in assessing them. As the β cell protein sequences differ between mice and humans, HIPs are usually not conserved across the two species. Gluten deamidation, on the other hand, should generate the same sequences in both. Citrullination is likely a mixed bag, as there is some conservation of sequences around citrullination sites. Moreover, the distinct MHC molecules in mice and humans also complicate being able to use mouse models to interrogate human epitopes. Therefore, for human studies, PBMCs are likely the most accessible source of T cells. Sampling of PBMCs can be used in all three cases, but there is no consensus on whether they are representative of the active pathogenic T cells from tissues [116, 213, 214]. Moreover, even in the most ideal cases, the frequency of antigen‐specific T cells within PBMCs is under 10%. This inherently limits identification of PTM‐reactive T cells from PBMCs to antigen‐directed methods.

## 7 Outlook for Antigen Discovery

For all three PTMs, if one is investigating known epitopes, synthetic peptides are still the method of choice, whether the starting material is tissues, PBMCs, or cloned TCRs. However, the method of choice when trying to identify novel epitopes or to deorphanize TCRs suspected to be PTM‐reactive differs greatly. For citrullination, proteomics or immunopeptidomics, followed by synthetic peptides, is currently the best approach. Higher‐throughput discovery methods are in development for citrullination and other PTMs by our group. For HIPs and deamidated epitopes, SABRs or similar methods that use encoded pMHC complexes would be appropriate, as these methods can perform large comprehensive screens. The epitopes identified with these methods will need to be followed up with synthetic peptide‐based validation assays.

Through the above examples, we have highlighted how discovery approaches for different PTMs differ. The technical and biological aspects of PTM biology and immunogenicity need to be considered when designing strategies for antigen discovery.

## 8 Summary and Future Outlook for Fishing for PTMs

Here, we have described key approaches for PTM antigen discovery. While synthetic peptides remain the most straightforward and commonly used method, newer methods including cell‐based methods are making rapid strides in their applicability to PTMs. With these methods taking the challenge of PTM antigen discovery head‐on, we believe that the landscape of PTM antigens will be increasingly accessible.

## Funding

This work was supported by National Institutes of Health, DP2OD033187, R01AT012826, R03DK127447, R21AI190660. Juvenile Diabetes Research Foundation United States of America, 3‐SRA‐2023‐1354‐S‐B. University of Pittsburgh.

## Conflicts of Interest

The authors declare no conflicts of interest.

## Data Availability

Data sharing not applicable to this article as no datasets were generated or analysed during the current study.
